# A Diffuse Fine Papular and Pustular Rash in a Man With Advanced Human Immunodeficiency Virus and Diabetes

**DOI:** 10.1093/cid/cix711

**Published:** 2018-01-18

**(See pages 477–8 for the Answer to the Photo Quiz.)**

**Figure 1.**
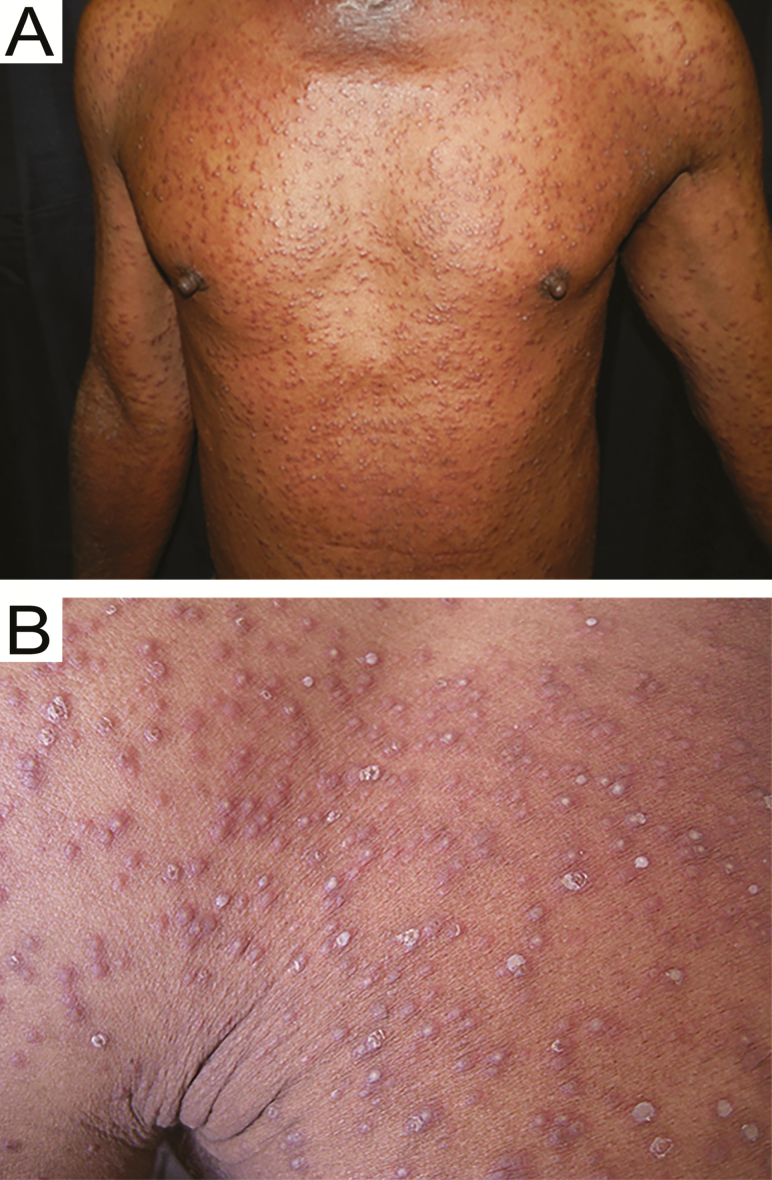
Extensive erythematous papules and pustules on the chest and arms (*A*) with close-up view around the shoulder (*B*).

**Figure 2.**
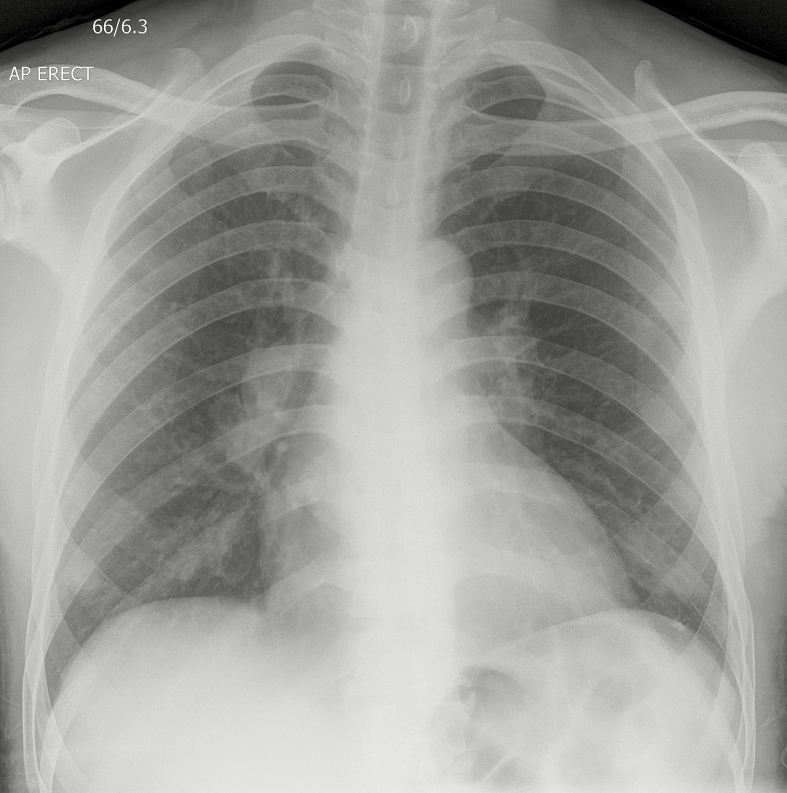
Chest radiograph with bilateral hilar lymphadenopathy and bilateral nodular pulmonary infiltrates.

**Figure 3.**
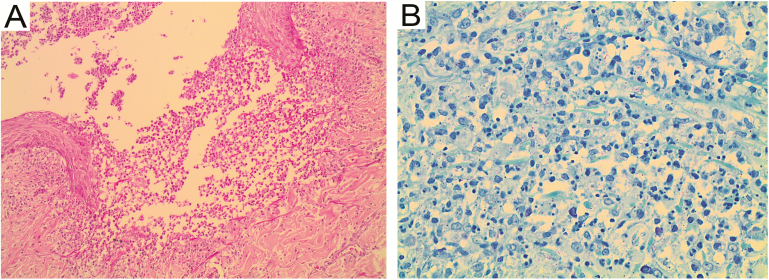
Skin punch biopsy showing subcorneal pustule with microabscess formation extending into the dermis (*A*). Acid-fast bacilli were seen on Ziehl-Neelsen staining (*B*).

A 63-year-old man was recently diagnosed with type 2 diabetes mellitus, for which he was taking metformin 1 g twice a day, and human immunodeficiency virus with a CD4 count of 5 cells/μL, for which he was not yet started on antiretroviral therapy. The skin lesions started 6 days prior on the chest, but rapidly spread to include the entire trunk, neck, face, and all limbs. He also reported a 2-week history of dry cough, with pleuritic chest pain, drenching night sweats, and mild loss of weight. On physical examination, there were extensive erythematous papules and pustules on the trunk and limbs (Figure 1), on a background of chronic eczematous changes in a photosensitive distribution. Laboratory studies revealed a normocytic anemia (hemoglobin 9.5 g/dL, mean corpuscular volume 85 fL). A chest radiograph (Figure 2) and skin punch biopsy (Figure 3) were performed.

What is your diagnosis?

